# Community- and Hospital-Acquired *Klebsiella pneumoniae* Urinary Tract Infections in Portugal: Virulence and Antibiotic Resistance

**DOI:** 10.3390/microorganisms7050138

**Published:** 2019-05-16

**Authors:** Cátia Caneiras, Luis Lito, José Melo-Cristino, Aida Duarte

**Affiliations:** 1 Microbiology and Immunology Department, Interdisciplinary Research Centre Egas Moniz (CiiEM), Faculty of Pharmacy, Universidade de Lisboa, 1649-003 Lisboa, Portugal; aduarte@ff.ulisboa.pt; 2 Institute of Environmental Health (ISAMB), Faculty of Medicine, Universidade de Lisboa, 1649-028 Lisboa, Portugal; 3 Laboratory of Microbiology, Centro Hospitalar Lisboa Norte, 1649-035 Lisboa, Portugal; lmlito@chln.min-saude.pt (L.L.); melo_cristino@medicina.ulisboa.pt (J.M.-C.); 4 Institute of Microbiology, Institute of Molecular Medicine, Faculty of Medicine, Universidade de Lisboa,1649-028 Lisboa, Portugal

**Keywords:** *Klebsiella pneumoniae*, multidrug resistance, virulence genes, urinary tract infections

## Abstract

*Klebsiella pneumoniae* is a clinically relevant pathogen and a frequent cause of hospital-acquired (HA) and community-acquired (CA) urinary tract infections (UTI). The increased resistance of this pathogen is leading to limited therapeutic options. To investigate the epidemiology, virulence, and antibiotic resistance profile of *K. pneumoniae* in urinary tract infections, we conducted a multicenter retrospective study for a total of 81 isolates (50 CA-UTI and 31 HA-UTI) in Portugal. The detection and characterization of resistance and virulence determinants were performed by molecular methods (PCR, PCR-based replicon typing, and multilocus sequence typing (MLST)). Out of 50 CA-UTI isolates, six (12.0%) carried β-lactamase enzymes, namely *bla*_TEM-156_ (n = 2), *bla*_TEM-24_ (n = 1), *bla*_SHV-11_ (n = 1), *bla*_SHV-33_ (n = 1), and *bla*_CTX-M-15_ (n = 1). All HA-UTI were extended-spectrum β-lactamase (ESBL) producers and had a multidrug resistant profile as compared to the CA-UTI isolates, which were mainly resistant to ciprofloxacin, levofloxacin, tigecycline, and fosfomycin. In conclusion, in contrast to community-acquired isolates, there is an overlap between virulence and multidrug resistance for hospital-acquired UTI *K. pneumoniae* pathogens. The study is the first to report different virulence characteristics for hospital and community *K. pneumoniae* pathogens, despite the production of β-lactamase and even with the presence of CTX-M-15 ESBL, a successful international ST15 clone, which were identified in both settings. This highlights that a focus on genomic surveillance should remain a priority in the hospital environment.

## 1. Introduction

The Gram-negative *Klebsiella pneumoniae* is a clinically relevant pathogen that has the propensity to acquire multidrug resistance (MDR), thus limiting the therapeutic options for treating related infections such as pneumonia, liver abscess, meningitis, bloodstream infections, and urinary tract infections (UTIs) [1].

*K. pneumoniae* is the second most frequent etiological agent involved in community-acquired (CA) UTIs [2,3], and it is one of the top three pathogens of international concern documented in the 2017 World Health Organization’s (WHO) Global Priority List of Antibiotic-Resistant Bacteria to Guide Research, Discovery, and Development of New Antibiotics [4]. 

Extended-spectrum β-lactamases (ESBL) are bacterial enzymes that confer resistance against a number of commonly used classes of β-lactam antibiotics [5]. The emergence of antimicrobial resistance, as *Klebsiella pneumoniae* carbapenemases (KPC) and cefotaximases (CTX-M) enzymes production, additional to the rapid worldwide spread of *K. pneumoniae* ESBL producers is posing a serious threat to global health [6], despite the implementation of local and national guidelines [7] as well as the development innovative approaches that are being considered for therapeutics [8]. 

*K. pneumoniae* utilizes a variety of virulence factors, especially capsule polysaccharides, adhesins, and determinants for iron acquisition, which are used for survival and immune evasion during infection [9]. Typically, *K. pneumoniae* is an opportunistic pathogen, which mostly affects those with weakened immune systems and tends to cause hospital-acquired (HA) infections [10]. However, virulent *K. pneumoniae* serotypes can lead to neonatal sepsis in immunocompromised patients [11], hospital intensive care unit patients [12], or previously healthy persons, and in all cases, it can cause life-threatening infections [13]. We previously reported a high prevalence of virulence determinants on multidrug-resistant sequence type (ST)14 KPC-3 carbapenemase *K. pneumoniae* [14] and detailed the possibility of this microorganism preceding difficult-to-treat and fatal infections, including those caused by other Gram-negative pathogens, such as *A. baumannii* [15]. However, despite the recent interest in this relationship, the interplay between resistance and virulence in *K. pneumoniae* clinical isolates remains poorly understood [9], and to our knowledge, the pathogenic potential of *K. pneumoniae* in urinary tract infections, especially in the community setting, and its resistance profile have not yet been characterized. 

Therefore, we conducted a multicenter retrospective study for a total of 81 isolates (50 CA-UTI and 31 HA-UTI) in Portugal with the main goal of investigating the virulence and antibiotic resistance of *K. pneumoniae* in urinary tract infections recovered from hospital and community clinic environments.

## 2. Materials and Methods

### 2.1. Bacterial Isolates

A total of 81 non-duplicated *K. pneumoniae* clinical isolates from urinary tract infections were studied. Only one isolate was considered per patient. The CA-UTI *K. pneumoniae* isolates (n = 50) were recovered from 10 community laboratories located in Portugal. Only *K. pneumoniae* isolates were selected for the study. The specimens were consecutively collected in the period from January to March 2010. The HA-UTI *K. pneumoniae* isolates (n = 31) were collected from hospitalized patients at a tertiary care university hospital center located in Lisbon between 1980 and 2013 and were selected from the Faculty of Pharmacy, University of Lisboa (FFUL) collection based on the source of isolation (urine only), by beta-lactamase type, and within this, by random selection. All uropathogens were obtained as part of routine care and were recovered under standard operating procedures. The identification of bacteria and β-lactamase production was primarily performed by microbiology laboratories using conventional methods or automated systems such as Vitek2^®^ (BioMérieux, Marcy, l’Étoile, France) or MicroScan^®^ (Snap-on, Kenosha, WI, USA). Thereafter, the bacteria were sent to the Faculty of Pharmacy, Department of Microbiology and Immunology Laboratory for specific molecular studies. The isolates were frozen in brain heart infusion (BHI) broth (VWR Prolabo, Lisboa, Portugal) with 15% glycerol at −80 °C. Bacterial growth was done using BHI broth (18 h, 37 °C) and the bacteria were later seeded in Luria–Bertani agar (VWR Prolabo^®^, Lisboa, Portugal).

### 2.2. Antimicrobial Susceptibility Testing and Phenotypic Detection of Extended-Spectrum β-lactamase (ESBL) Production

Antimicrobial susceptibility testing was performed using the standardized Kirby–Bauer disk diffusion technique, in accordance with the European Committee on Antimicrobial Susceptibility Testing (EUCAST) for Antimicrobial Susceptibility Testing guidelines; the detailed methodology is available at http://www.eucast.org/ast_of_bacteria/disk_diffusion_methodology/. Detailed instructions for Mueller–Hinton agar medium (VWR Prolabo, Lisboa, Portugal), including preparation and storage, are also available in the same EUCAST guidelines document. Quality control strains were included to monitor the performance of the test. The *K. pneumoniae* clinical isolates was tested for their susceptibility to the following antimicrobial agents: amoxicillin/clavulanic acid (20 µg/10 µg), ceftazidime (10 µg), cefotaxime (5 µg), cefoxitin (30 µg), ciprofloxacin (5 µg), levofloxacin (5 µg), gentamicin (10 µg), imipenem (10 µg), meropenem (10 µg), ertapenem (10 µg), tigecycline (15 µg), and fosfomycin (200 µg). The inhibition zones were interpreted in accordance with EUCAST, except for fosfomycin, which was interpreted using breakpoints proposed by the Clinical and Laboratory Standard Institute guidelines [16]. Zone diameters of susceptibility categories were formed according to the proposed definitions of the 2018 EUCAST Steering Committee [17], which are available at http://www.eucast.org; namely, categories included (1) susceptible, standard dosing regimen (S); (2) susceptible, increased exposure (I); (3) and resistant (R). Multidrug resistance (MDR) was defined as non-susceptibility to at least one agent in three or more antimicrobial categories [18].

The phenotypic double-disk synergy test (DDST), which involves the application of disks containing ceftazidime next to a disk with amoxicillin–clavulanic acid on Mueller–Hinton agar plates, was performed on the *K. pneumoniae* uropathogens that showed resistance to at least one of the tested third-generation cephalosporins (cefotaxime, ceftazidime). The methodology and interpretation of results was done according to the EUCAST guidelines for the detection of resistance mechanisms and specific resistances of clinical and/or epidemiological importance, which are available at http://www.eucast.org/resistance_mechanisms/.

### 2.3. Detection of Resistance and Virulence Genes

Isolates were screened by the polymerase chain reaction (PCR) technique using specific primers for the detection of β-lactamase-associated genes (*bla*_DHA_, *bla*_CMY_, *bla*_CTX-M_, *bla*_SHV_), including carbapenemase genes (*bla*_VIM_, *bla*_IMP_, *bla*_OXA_, and *bla*_KPC_). Moreover, the presence of the following K2 capsule serotypes (*K2A*) were also investigated; type 1 and type 3 fimbrial adhesins (*fimH* and *mrkD*, namely the subtypes *mrkD_V1_* and *mrkD_V2-4_*); siderophore aerobactin (*iucC*); haemolysin (*khe*); regulator of mucoid phenotype A (*rmpA*); and hypermucoviscosity phenotype (*magA*) virulence genes. Among the 79 known capsular serotypes of *K. pneumoniae*, K1 and K2 have been shown to be the most prevalent [19] and clinically relevant, despite their association with invasive infections [20,21,22]. The search for only the K2 capsular type was justified considering that K1 is mainly related to hypervirulent *K. pneumoniae* variants (hvKP), which cause pyogenic liver abscesses and other infections [23] and are mainly found in Asian countries [21], while K2 is usually related to non-hvKP isolates, which cause severe and difficult-to-treat infections [21,24,25]. Additionally, hypervirulent *K. pneumoniae* isolates are defined by either capsular type K1 or K2 (which confers different virulence characteristics such as serum resistance) or the presence of one of the following virulence genes: aerobactin (iuc), rmpA/rmpA2, and/or salmochelin (iro) [23].

The PCR methodology, including primer sequences, lengths of expected PCR products, quality control strains, as well the purification technique and nucleotide sequences analysis were previously described in detail [14].

### 2.4. Multilocus Sequence Typing

A total of twenty-four isolates, namely the eleven KPC-3 carbapenemase producers and thirteen CTX-M-15 cefotaximase extended-spectrum beta-lactamase-producing *K. pneumoniae* isolates (12 HA-UTI and 1 CA-UTI) were selected for multilocus sequence typing (MLST) after sequencing (Macrogen, Inc., Korea). Primers, PCR reaction conditions, and detailed methodology were in accordance with those previously described by Diancourt, L. et al. [26]. The allele attribution and sequence type (ST) identification was done with the *K. pneumoniae* MLST database platform from Institute Pasteur (http://www.pasteur.fr/mlst/; Last accessed on 2 May 2018).

### 2.5. PCR-based Replicon Typing

The molecular identification and classification of plasmids, namely the identification of origins of plasmid replicates belonging to different incompatibility groups, was performed by the PCR-based replicon typing (PBRT) technique described by Carattoli, A. et al. [27].

### 2.6. Statistical Analysis

The statistical analysis used the Fisher’s exact test, using the computer program available at http://www.graphpad.com/quickcalcs/index. A probability value of *p* ≤ 0.05 was considered to indicate statistical significance.

### 2.7. Ethical Approval

The Ethics Committee of the Faculty of Medicine, Universidade de Lisboa approved this study proposal. All isolates were recovered as part of routine testing, studied anonymously, and the epidemiological data were obtained retrospectively from clinical records.

## 3. Results

### 3.1. Antimicrobial Susceptibility

**Figure 1:**
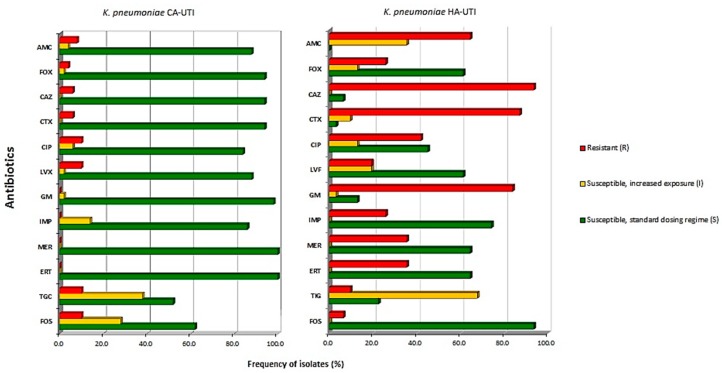
Antimicrobial susceptibilities of the *K. pneumoniae* clinical isolates (n = 81). The isolates were recovered from community-acquired (CA)-urinary tract infection (UTI) and hospital-acquired (HA)-UTI patients. The CA isolates were consecutively collected while the HA isolates were selected from the FFUL collection based on the source of isolation (urine only), beta-lactamase type produced, and within this, by random selection. Legend: AMC, amoxicillin/clavulanic acid; FOX, cefoxitin; CAZ, ceftazidime; CTX, cefotaxime; CIP, ciprofloxacin; LVF, levofloxacin, GM, gentamicin; IMP, imipenem; MER, meropenem; ERT, ertapenem; TIG, tigecycline; FOS, fosfomycin; FFUL, Faculty of Pharmacy University of Lisboa.

The susceptibility of the CA-UTI *K. pneumoniae* isolates was found to be 100% (50/50) to meropenem and ertapenem, 98% (49/50) to gentamicin, 94% (47/50) to ceftazidime, cefoxitin, and cefotaxime, 88% (44/50) to amoxicillin/clavulanic acid and levofloxacin, 86% (43/50) to imipenem, and 84% (42/50) to ciprofloxacin. Higher resistance rates were found for ciprofloxacin, levofloxacin, tigeciclin, and fosfomycin with 10% (5/50) resistant isolates for each. Fourteen percent (7/50), 28% (14/50), and 38% (19/50) of the isolates belonged to category I (susceptible, increased exposure) for imipenem, fosfomycin, and tigeciclin, respectively. The results are shown in Figure 1. On the other hand, the HA-UTI *K. pneumoniae* isolates showed a full antimicrobial resistance profile, namely, 93.5% (29/31), 87.1% (27/31), and 64.5% (20/31) of the isolates were resistant to ceftazidime, cefotaxime, and amoxicilin/clavulanic acid, respectively. Additionally, 83.9% (26/31) and 41.9% (13/31) of the isolates were resistant to gentamicin and ciprofloxacin, respectively. The most active antibiotics were shown to be fosfomycin (93.5%, 20/31), imipenem (74.2%, 23/31), and meropenem, and ertapenem (64.5%, 20/31 each). Imipenem and fosfomycin were found to be non-susceptible, increased exposure (I) isolates.

### 3.2. Identification of the β-Lactamases

PCR amplification of β-lactamase genes was performed in CA-UTI and HA-UTI *K. pneumoniae* isolates. ESBL production was detected in six (12.0%) CA-UTI *K. pneumoniae* isolates, namely, bla_TEM-156_ (n = 2), bla_TEM-24_ (n = 1), bla_SHV-11_ (n = 1), bla_SHV-33_ (n = 1), and bla_CTX-M-15_ (n = 1). The results are presented in Table 1. The multidrug resistant HA-UTI *K. pneumoniae* isolates carried the ESBLs bla_TEM-10_ (19.3%, 6/31), bla_TEM-24_ (9.6%, 3/31), bla_CTX-M-15_ (38.7%, 12/31), bla_KPC-3_ (19.3%, 6/31), bla_KPC-3_ and bla_SHV-11_ (3.2%, 1/31), and bla_KPC-3_ and bla_CTX-M-15_ (12.9%, 4/31). The genes *bla_DHA_*, *bla_CMY_*, *bla_IMP_*, *bla_VIM_*, *bla_NDM_*, and *bla_OXA_* were not detected in our study. The replicon typing plasmids found are also presented in Table 1. The conjugative plasmid IncFIA was the most commonly found (66.7%, 4/6) CA-UTI *K. pneumoniae* β-lactamase producer, while HI1 (CTX-M-15 producers), A/C, and F (KPC-3 producers) were mainly present (38.7% 12/31, 22.6% 7/31, and 25.8% 8/31, respectively) on the HA-UTI *K. pneumoniae* isolates The plasmids were non-typeable by the former scheme among eight isolates.

### 3.3. Virulence Genes

Of all the *K. pneumoniae* UTI clinical isolates (n = 81), whose results are shown in Table 2, the major virulence genes identified were *fimH* (63%, 51/81), *mrkD_V1_* (49.3%, 40/81), and *khe* (60.5%, 49/81). A total of 38.2% (31/81) of the isolates showed the presence of *mrkD_V2-4_*, while 18.5% (15/81) and 16.0% (13/81) showed the *iucC* and *K2A* genes, respectively. No *magA* and *rmpA* genes were amplified. The type 3 fimbrial adhesin *mrkD_V1_* was predominant in HA-UTIs (96.8%, 30/31) but not in CA-UTIs (20.0%, 10/50), while *mrkD_V2-4_* was the most common type 3 fimbrial adhesin (62.0%, 31/50 vs. 20.0% 10/50) in CA-UTIs, and it was only found in these isolates. Additionally, higher virulence potential was found in the HA-UTI isolates with an average of 3.29 virulence factors per isolate in HA-UTI vs. 1.94 virulence factors per isolate in CA-UTI.

In order to identify how the virulence genes were simultaneously present on the same isolate, we characterized the virulence profiles (VP) of both CA- and HA-UTI *K. pneumoniae* uropathogens. The results are presented in Table 3.

A total of 24 different virulence profiles was identified in our study (Table 3). The most frequent virulence profile found was VP13 (*fimH, khe, mrkD_V1_*), which was observed in 18.5% (15/81) of the isolates, but mainly in HA-UTI isolates (40.6% HA-UTI vs. 4.0% CA-UTI, *p* < 0.05). A similar finding was obtained for VP22 (*K2, fimH, khe, mrkD_V1_*) which showed a prevalence of 8.6% (7/81) but was only found in HA-UTI isolates (21.9%, 7/31). Similarly, virulence profiles with no virulence genes (9.9%, 8/81) and one virulence factor, namely VP2 with the virulence factor *khe*, accounted for 6.2% (5/81) of the isolates, and this type of profile was only found in CA-UTI *K. pneumoniae* isolates. All HA-UTI isolates harbored at least one virulence gene. Moreover, the HA-UTIs presented only nine virulence profiles (VP 13 and VP 22 with two and four virulence genes, respectively, accounted for 62.5% of the isolates) while CA-UTI virulence profiles were distributed among 18 virulence profiles, and the most frequent profiles, VP 1 and VP2, presented 0 and 1 virulence factors, respectively, and accounted for only 26.0% of the CA-UTI isolates.

### 3.4. MLST Results

The clonal relatedness of the MDR CTX-M-15 ESBL *K. pneumoniae* producers, including one isolate identified in the community setting, was investigated by multilocus sequence typing (MLST). CTX-M-15 was included due to its clinical relevance to human health and because it is the only extended-spectrum-β-lactamase shared between hospital and community settings. The ST15 clone was identified in all CTX-M-15 isolates studied (100.0%, 13/13).

## 4. Discussion

Multidrug resistance and virulence are typically observed in separate *K. pneumoniae* populations [28] and their interplay remains poorly understood [9]. Recent reports have shown that *K. pneumoniae* strains can accumulate, increasing their pathogenicity and causing severe and difficult-to-treat infections. However, the available reports mainly cover the hypervirulent phenotypes of *K. pneumoniae* (mainly related to pyogenic liver abscesses and meningitis [28,29]), focus on carbapenemase producers [30], and were performed in the hospital setting [14,25]. Our study characterizes the virulence and antibiotic resistance traits of *K. pneumoniae* in urinary tract infections recovered from both community and hospital settings. Additionally, this is the first report of a community-acquired urinary tract infection related to the CTX-M-15 ESBL *K. pneumoniae* producer, which belongs to the successful international ST15 clone, in Portugal.

The majority of CA-UTI *K. pneumoniae* pathogens characterized in our study were susceptible isolates; the antimicrobial susceptibility was >80% in eight out of ten tested antibiotics. However, the number of isolates belonging to category I (susceptible, increased exposure) found for imipenem (14%) deserves particular attention considering that the isolates were inhibited in vitro by a concentration of antibiotics that is associated with an uncertain therapeutic effect. In Portugal, carbapenems consumption is increasing [31]. Portugal is now one of the top consumers in Europe, and resistance to antibiotics of this class has changed from 5.2% in 2016 [32] to 8.6% in 2017 [33] according to the resistance surveillance network (EARS-Net) of the European Centre for Disease Prevention and Control (ECDC). Strict monitoring of imipenem consumption and continuous monitoring of evolutionary trends in the susceptibility patterns of *K. pneumoniae*, especially regarding community-acquired infections, is mandatory.

Limited data are available on the susceptibility to recently accessible antimicrobial agents, such as tigecycline, along with some “older” antibiotics, such as fosfomycin, particularly on multidrug and ESBL-producing Gram-negative microorganisms [34]. The in vitro activities of these antibiotics were determined against *K. pneumoniae* isolates recovered from community- and hospital-acquired urinary tract infections. Surprisingly, a significantly lower susceptibility (62% CA-UTI vs. 94% MDR HA-UTI) to the fosfomycin antibiotic was found for CA-UTI *K. pneumoniae* isolates when compared with HA-UTIs. Similarly, susceptibility rates of 96% for MDR *Enterobacteriaceae* [35] and 89% for *K. pneumoniae* KPC producers were found [14], including a report showing that extremely drug-resistant *K. pneumoniae* KPC producers, including tigecycline and colistin resistant types, were susceptible to fosfomycin [36]. However, lower fosfomycin activity has been previously reported, perhaps due to differences in local epidemiology [29]. The present study demonstrates that fosfomycin, an older antimicrobial agent, should be considered to be an emerging treatment option for difficult-to-treat hospital-acquired urinary tract infections caused by *K. pneumoniae* uropathogens, including MDR isolate multidrugs, but caution should be applied for its use as an alternative agent for outpatient therapy of UTIs.

The presence of virulence factors has an important contribution to the pathogenesis of *K. pneumoniae* [13] and to the development of severe and invasive forms of infection, not only in immunocompromised individuals but also in previously healthy adults [11,25,37]. Bandeira et al. support the hypothesis that biofilms formed on medical devices can promote the onset and spread of healthcare-associated infections, and they reported that biofilm-forming bacteria are generally more resistant to antibiotics [37]. In our study, the most frequent virulence factors found were *fimH* and the *mrkD*, which encode type 1 and type 3 fimbrial adhesins, respectively, which mediate binding to epithelial cells of the urinary tract and promote biofilm development [38,39,40]. In fact, despite the findings of the present study being similar to those of other studies that have reported the ubiquitous nature of these fimbriae in *K. pneumoniae* [40,41], a significantly higher prevalence of fimbriae on HA uropathogens when compared with CA isolates was found in our study. This could lead to the infection of medical devices and may explain their persistence and difficulty of eradication in the hospital setting.

The genes involved in the synthesis of siderophores have dual roles as they can also inhibit T cell proliferation, promoting host immunosuppression, in addition to their role in enhancing iron uptake [42]. The iron siderophore aerobactin synthase gene (*iucC*) was detected in 19% of the isolates and showed similar prevalence between CA and HA uropathogens. Despite the prevalence found in our study being lower than that previously reported (32%) [25], it is unusual to have high identification of community-provenance isolates. The *rmpA* and *magA* genes were not found in our uropathogens. These genes are frequently identified in *K. pneumoniae* causing liver abscesses, particularly in Asian countries [43,44]. The capsule polysaccharide K2 serotype has been previously reported as a major contributor to the virulence of *K. pneumoniae* isolates [45]. It confers resistance to phagocytosis [46] and is related to severe infections [47]. In our study, 16.0% (13/81) of the isolates showed the K2 capsular serotype, mainly MDR HA uropathogens. Despite the results being in accordance with previous reports [45], unusual capsular types have been described in carbapenem-resistant *K. pneumoniae* strains, such as K64 and K62. [48]. Therefore, future studies on virulence should consider the characterization of capsular types for *Klebsiella* spp. strains [47].

Differences between uropathogens from the hospital and community settings were also found in terms of the accumulation of virulence genes by each isolate. The *K. pneumoniae* recovered from the hospital setting showed higher pathogenic potential, with higher genomic complexity and adaptation to two specific profiles. These findings have important clinical relevance, considering that the presence of virulence factors in *K. pneumoniae* has been previously described as the most prominent cause of death in patients before starting antibiotic therapy [49]. The virulence profiles VP13 (*fimH, khe, mrkD_V1_*) and VP22 (*K2, fimH, khe, mrkD_V1_*) were predominant in the hospital-acquired collection. The accumulation of type 1 and type 3 fimbrial adhesins with haemolysin, a toxin that has cytolytic and cytotoxic activity against a wide range of mammalian cell types [50], seems to be an effective virulence combination. Additionally, it is notable that both major virulence profiles identified in HA-UTIs had the same virulence genes, with or without the presence of capsular serotype K2.

High diversity and low accumulation of virulence genes in the same isolate was identified in *K. pneumoniae* community uropathogens, reinforcing the low pathogenicity of these isolates reported in our study.

A proficient pathogen should be virulent, resistant to antibiotics, and epidemic [9]. Genotyping of the multidrug-resistant CTX-M-15-producing *K. pneumoniae* isolates by MLST recovered the ST15 clone in isolates from both hospital and community settings. Of relevance, no associations between the ST15 MLST type, antimicrobial resistance, and virulence profiles, or between the ESBLs produced and the virulence profiles, were found in our study. However, there was a clear accumulation of virulence factors in highly multidrug resistant *K. pneumoniae* clinical isolates recovered from hospital-acquired urinary tract infections when compared with those recovered from the community setting, which showed low resistance and a low virulent potential profile. These results indicate a direct relationship and relevant clinical interplay between resistance and virulence in *K. pneumoniae* clinical isolates, and the supremacy of antimicrobial resistance appears to be the leading factor.

In 2018, we reported that the ST11 that belongs to the CC258 group, one of the most threating MDR Gram-negative bacterias circulating in nosocomial settings worldwide [51], has been replaced by the ST14 clone KPC-3 carbapenemase-producing *K. pneumoniae* isolates [14]. In the present study we report the identification of the ST15 clone of CTX-M-15-producing *K. pneumoniae* in both the hospital and community settings and demonstrate that this clone can accumulate virulence and MDR. ST15 is described as a successful international clone that is present worldwide and has recently been associated with colistin-resistant infections [52]. It is highly virulent and resistant [53] and was one of the first *K. pneumoniae* isolates in the United States to be reported to the Centers for Disease Control and Prevention (CDC) as non-susceptible to all drugs tested, including all beta-lactams, colistin, and tigecycline [54]. A limitation of the present study is that we showed only a small part of the real epidemiological situation considering the collection studied. Also, despite being a multicenter study, not all districts of Portugal were included, and the period of study can be considered too large for a short number of isolates. Additionally, only CTX-M-15 and KPC-3 producers were characterized by MLST. Future studies should expand the collection of pathogens tested and the number of centers involved in order to provide a more complete picture of the dissemination of *K. pneumoniae* in Portugal. However, a complete microbiological and molecular characterization of *K. pneumoniae* strains collected from urinary tract infections was performed, including antimicrobial resistance profiling, ESBL characterization, and detection of virulence determinants. Thus, the results found in this study have great importance from both the clinical and research points of view.

## 5. Conclusions

In conclusion, there is an overlap between virulence and multidrug resistance in MDR hospital-acquired UTI *K. pneumoniae* pathogens but not in community-acquired isolates. Different virulence characteristics were reported, despite the production of β-lactamase and even in the presence of the successful international CTX-M-15 ESBL ST15 clone in both settings. These results highlight that the genomic surveillance focus should remain a priority in the hospital environment.

## Figures and Tables

**Table 1:** Antibiotic resistance profile, distribution of virulence genes, and plasmid content of β-lactamases-producing *K. pneumoniae* community-acquired UTI isolates.

	Isolate ID	Date of Isolation	β-Lactamase Produced	Antibiotics Resistance Profile	Virulence Profile	PBRT	MLST
***CA-UTI isolates***	201-010	2010	TEM-156	CIP, LVF	*khe, mrkD_V1_*	FIA	NP
	201-075	2010	SHV-11	CIP, FOS	*mrkD_V2-4_*	FIA, X, Y	NP
	201-076	2010	TEM-24	LVF, FOS	*No VG*	X, A/C	NP
	201-094	2010	TEM-156	AMC, FOS	*mrkD_V1_*	W	NP
	208-309	2010	SHV-33	NR	*fimH, mrkD_V2-4_, iucC*	FIA, Y	NP
	212-193	2010	CTX-M-15	AMC, CAZ, CTX, CIP, LVF	*khe, iucC*	FIA, HI2	ST15
***HA-UTI isolates***	625	1980	TEM-10	GM	*K2, fimH, khe, mrkD_V1_*	NT	NP
	683	1999	TEM-10	AMC, CTX, CAZ, GM	*K2, fimH, khe, mrkD_V1_*	NT	NP
	684	1999	TEM-10	AMC, CAZ, GM	*K2, fimH, khe, mrkD_V1_*	NT	NP
	712	1999	TEM-24	AMC, CTX, CAZ	*fimH, khe, mrkD_V1_*	NT	NP
	721	1999	TEM-10	AMC, CTX, CAZ, GM	*fimH, khe, mrkD_V1_*	NT	NP
	732	2000	TEM-10	AMC, CTX, CAZ, GM	*fimH, khe, mrkD_V1_*	NT	NP
	749	2001	TEM-24	AMC, FOX, CTX, CAZ, CIP	*fimH, khe*	NT	NP
	770	2001	TEM-24	AMC, FOX, CTX, CAZ, CIP	*fimH, khe*	NT	NP
	775	2001	TEM-10, CTX-M-15	CAZ, GM	*fimH, khe, mrkD_V1_*	HI1	ST15
	847	2003	CTX-M-15	AMC, CTX, CAZ, GM, CIP	*fimH, khe, mrkD_V1_*	HI1	ST15
	931	2004	CTX-M-15	CTX, CAZ, GM, CIP	*fimH, khe, mrkD_V1_*	HI1	ST15
	1119	2005	CTX-M-15	CTX, CAZ, GM, CIP	*fimH, khe, mrkD_V1_*	HI1	ST15
	1263	2007	CTX-M-15	GM, CIP	*fimH, khe, mrkD_V1_*	HI1	ST15
	2323	2008	CTX-M-15	AMC, CTX, CAZ, GM	*fimH, khe, mrkD_V1_*	HI1	ST15
	2325	2008	CTX-M-15	AMC, CTX, CAZ, GM	*fimH, mrkD_V1_*	HI1	ST15
	2386	2008	CTX-M-15	CTX, CAZ, GM	*fimH, khe, mrkD_V1_*	HI1	ST15
	2394	2008	CTX-M-15	CTX, CAZ, GM	*fimH, khe, mrkD_V1_*	HI1	ST15
	2398	2008	CTX-M-15	CTX, CAZ, GM, CIP	*fimH, mrkD_V1_*	HI1	ST15
	2400	2008	CTX-M-15	CTX, CAZ, GM	*fimH, khe, mrkD_V1_*	HI1	ST15
	2414	2008	CTX-M-15	CTX, CAZ, GM, CIP	*fimH, khe, mrkD_V1_*	HI1	ST15
	2909	2009	KPC-3, SHV-11	AMC, CTX, CAZ, IMP, GM, MEM, ERT	*K2, fimH, khe, mrkD_V1_*	F	ST14
	2954	2010	KPC-3	AMC, FOX CTX, CAZ, IMP, GM, LVF, TIG, MEM, ERT	*K2, fimH, khe, mrkD_V1_*	A/C, F	ST14
	3108	2010	KPC-3	AMC, CTX, CAZ, TIG, MEM, ERT	*khe, mrkD_V1_, iucC*	FIA, F	ST14
	29078	2010	KPC-3, CTX-M-15	AMC, FOX, CTX, CAZ, IMP, GM, MEM, ERT, FOS	*fimH, khe, mrkD_V1_,iucC*	FIA, F	ST14
	43201	2010	KPC-3, CTX-M-15	AMC, FOX, CTX, CAZ, IMP, GM, CIP, LVF, MEM, ERT	*K2, fimH, khe, mrkD_V1_,iucC*	A/C	ST14
	61095	2011	KPC-3, CTX-M-15	AMC, CTX, CAZ, IMP, GM, CIP, LVF, MEM, ERT, TIG	*K2, fimH, mrkD_V1_*	A/C, F	ST14
	72562	2011	KPC-3	AMC, FOX, CTX, CAZ, MEM, ERT	*fimH, mrkD_V1_,iucC*	A/C	ST14
	87582	2012	KPC-3	AMC, FOX, CTX, CAZ, IMP, GM, CIP, LVF, MEM, ERT, FOS	*fimH, khe, mrkD_V1_,iucC*	F	ST14
	91488	2012	KPC-3, CTX-M-15	CTX, CAZ, GM, CIP, LVF, MEM, ERT	*K2, fimH, khe, mrkD_V1_*	A/C, F	ST14
	22073	2013	KPC-3	AMC, FOX, CTX, CAZ, IMP, GM, CIP, LVF, MEM, ERT	*K2, fimH, khe, mrkD_V1_*	A/C	ST14
	31149	2013	KPC-3	AMC, CTX, CAZ, IMP, GM, MEM, ERT	*K2, fimH, mrkD_V1_,iucC*	A/C, F	ST14

Legend: CA-UTI, community-acquired urinary tract infection; HA-UTI, hospital-acquired urinary tract infection; ID, identification; Nr., number; No VG; no virulence genes found; NR, no resistance found; CIP, ciprofloxacin; LVF, levofloxacin; AMC, amoxicillin/clavulanic acid; FOS, fosfomycin; CAZ, ceftazidime; CTX, cefotaxime; FOX, cefoxitin; GM, gentamicin; IMP, imipenem; MER, meropenem; ERT, ertapenem; TIG, tigecycline; PBRT, PCR-based replicon typing; NT, non-typeable; NP, not performed; MLST, multilocus sequence typing; ST, sequence type; KPC, *Klebsiella pneumoniae* carbapenemase; CTX, cefotaximase.

**Table 2:** Distribution of virulence genes in *K. pneumoniae* isolates from community-acquired urinary tract infections (CA-UTI) and hospital-acquired urinary tract infections (HA-UTI).

Virulence Factor		Target Gene	Nr. of CA-UTI Isolates (%)	Nr. of HA-UTI Isolates (%)	Total (%)
			(n = 50)	(n = 31)	(n = 81)
***Fimbrial adhesins***	*Type 1*	*fimH*	20 (40.0)	31 (100.0) *	51 (63.0)
	*Type 3, variant 1*	*mrkD_V1_*	10 (20.0)	30 (96.8) *	40 (49.3)
	*Type 3, variant 2-4*	*mrkDV_2-4_*	31 (62.0)	0 *	31 (38.2)
***Toxin***	*Haemolysin*	*khe*	23 (46.0)	26 (83.9) *	49 (60.5)
***Capsular type***	*K2 serotype*	*K2A*	3 (6.0)	10 (32.2) *	13 (16.0)
***Siderophore***	*Aerobactin*	*iucC*	10 (20.0)	5 (16.1)	15 (18.5)
***Protectins or invasins***	*Mucoviscosity phenotype*	*magA*	0	0	0
	*Regulator of mucoid phenotype*	*rmpA*	0	0	0

Legend: CA-UTI, community-acquired urinary tract infection; HA-UTI, hospital-acquired urinary tract infection; Nr., number; * *p*-values less than 0.05 were considered statistically significant.

**Table 3:** Characterization of virulence profiles of community- and hospital-acquired UTI *K. pneumoniae* isolates.

Nr. of Virulence Genes	Virulence Profile (VP)	Virulence Genes	Nr. Isolates (%)		
			CA-UTI n = 50 (100)	HA-UTI n = 31 (100)	Total n = 81 (100)
0 VF	1	*No VF*	8 (16.0)	0	8 (9.9)
1 VF	2	*khe*	5 (10.0)	0	5 (6.2)
	3	*fimH*	1 (2.0)	0	1 (1.2)
	4	*mrkD_V1_*	2 (4.0)	0	2 (2.5)
	5	*mrkD_V2-4_*	3 (6.0)	0	3 (3.7)
2 VF	6	*fimH, khe*	2 (4.0)	2 (6.3)	4 (4.9)
	7	*fimH, mrkD_V1_*	0	3 (9.4)	3 (3.7)
	8	*khe, iucC*	1 (2.0)	0	1 (1.2)
	9	*iucC, mrkD_V2-4_*	2 (4.0)	0	2 (2.5)
	10	*khe, mrkD_V1_*	2 (4.0)	0	2 (2.5)
	11	*khe, mrkD_V2-4_*	5 (10.0)	0	5 (6.2)
	12	*fimH, mrkD_V2-4_*	5 (10.0)	0	5 (6.2)
	13	*fimH, khe, mrkD_V1_*	2 (4.0)	13 (40.6)	15 (18.5)
	14	*fimH, khe, iucC*	1 (2.0)	0	1 (1.2)
	15	*fimH, khe, mrkD_V2-4_*	2 (4.0)	0	2 (2.5)
3 VF	16	*fimH, iucC, mrkD_V1_*	2 (4.0)	1 (3.1)	3 (3.7)
	17	*fimH, iucC, mrkD_V2-4_*	4 (8.0)	0	4 (4.9)
	18	*khe, mrkD_V1_, iucC*	0	1 (3.1)	1 (1.2)
	19	*K2, khe, mrkD_V1_*	2 (4.0)	0	2 (2.5)
	20	*K2, fimH, mrkD_V1_*	0	1 (3.1)	1 (1.2)
	21	*K2, fimH, khe, mrkD_V2-4_*	1 (2.0)	0	1 (1.2)
4 VF	22	*K2, fimH, khe, mrkD_V1_*	0	7 (21.9)	7 (8.6)
	23	*fimH, khe, mrkD_V1_, iucC*	0	2 (6.3)	2 (2.5)
5 VF	24	*K2, fimH, khe, mrkD_V1_, iucC*	0	1 (3.1)	1 (1.2)

Legend: VF, virulence factor; VP, virulence profile; CA-UTI, community-acquired urinary tract infection; HA-UTI, hospital-acquired urinary tract infection.
